# Leisure time physical activity in 9- to 11-year-old children born moderately preterm: a cohort study

**DOI:** 10.1186/s12887-018-1141-8

**Published:** 2018-05-12

**Authors:** M. Nordvall-Lassen, H. K. Hegaard, C. Obel, M. S. Lindhard, M. Hedegaard, T. B. Henriksen

**Affiliations:** 1 The Research Unit of Women’s and Children’s Health, section 7821, The Juliane Marie Centre, Copenhagen University Hospital, Rigshospitalet, Blegdamsvej 9, DK-2100 Copenhagen, Denmark; 2 Faculty of Health and Medical Sciences, University of Copenhagen, Copenhagen, Denmark; 3 Obstetric Clinic, The Juliane Marie Centre, Rigshospitalet, Copenhagen University Hospital, Copenhagen, Denmark; 4 Child, Family and Reproductive Health, Department of Health Science, Faculty of Medicine, Lund University, Lund, Sweden; 5 Institute of General Medical Practice, Department of Public Health, Aarhus University, Aarhus, Denmark; 6 Perinatal Epidemiology Research Unit, Department of Paediatrics, Aarhus University Hospital, Skejby, Aarhus, Denmark

**Keywords:** Preterm birth, Leisure time physical activity, Moderately preterm, School age children

## Abstract

**Background:**

Physical activity is one of the best documented activities with impacts on health in children and adults. Children born preterm show reduced physical and psychosocial function compared to children born at term. This may influence their level of physical activity. Reports on moderately preterm children’s physical activities during childhood are limited. Thus, the aim of this study was to compare the leisure time physical activity at age 9-11 years of moderately preterm children with that of children born at term.

**Methods:**

Data from 4941 mother-child pairs from the Aarhus Birth Cohort (1989-91) were used. The cohort gathered clinical information, including gestational age at delivery. Information about parental socio-demographic and lifestyle factors was obtained from questionnaires completed during the second trimester of pregnancy. Information about children’s physical activities was reported in a 9- to 11-year follow-up questionnaire completed by parents detailing how many times per week their child participated in sports activities outside of school, hours spent per week playing outside, and hours per week engaged in sedentary activities. Data were analysed using multiple logistic regression with the lowest activity group as a reference group.

**Results:**

A total of 158 children (3.2%) were born moderately preterm, i.e., between 32 and 36 completed weeks. Children born moderately preterm participated in sports activities as often as their peers born at term; they also participated in frequent sports activities (≥ 4 times per week) as often as their peers. There were no differences in hours per week spent playing outside or in sedentary activities between the two groups.

**Conclusions:**

Nine- to 11-year-old moderately preterm children participated in sports activities outside school to a similar extent as their peers and engaged in outdoor activities and sedentary activities for the same duration of time per week as their peers born at term.

## Background

Preterm delivery, defined as birth before 37 weeks of gestation [1], is strongly associated with neonatal morbidity and mortality [2] and with long-term sequelae [3–6]. Due to advances in neonatal intensive care, the survival of preterm children has increased considerably [7–10]. Preterm children are predisposed to difficulties with cognitive, behavioural, social, psychological, and motor functions [7, 11–16]; even moderately preterm (32-36 weeks) children are at an increased risk for developmental delay, neurologic impairments and learning problems [17, 18], which may contribute to difficulties participating in physical activities. Many studies have focused on these functional parameters, but little is known about normal everyday life functions, such as physical activity levels. Studies on adults with a birth weight of less than 1500 g have shown that they engage less frequently in leisure time physical activity [19, 20].

However, few studies have focused on physical activity in children born moderately preterm, even though this group makes up 80% of all premature births [17]. Physical inactivity is a risk factor for obesity, cardiovascular disease, type-2 diabetes, metabolic syndrome, and several other diseases [21, 22]. Adults born preterm have higher levels of risk factors for these diseases, such as high blood pressure [23], impaired glucose regulation [24, 25] and lower bone mineral density [26]. Cardiovascular disease risk factors have been reported to be reduced by regular physical activity, particularly in subjects with a low birth weight [27].

Research has shown that there is a life-trajectory of physical activity that begins early in life [28]. It is therefore important to investigate the level of physical activity in school-aged children who were born preterm without evident morbidity and who attend the same schools as children born at term. Do these children require special intervention to attain an adequate level of physical activity? Physical activity is quite possibly one of the best documented healthy activities for children [29]. The Danish National Board of Health has recommended a daily minimum of 60 min of moderate to high intensity physical activity and high intensity physical activity for a minimum 30 min at least three times a week for children [29].

Accordingly, we compared the leisure time physical activity of 9- to 11-year-old former moderately preterm children with their peers born at term using a birth cohort with a decade of follow-up. Moderately preterm delivery is defined as children born between 32 and 36 completed gestational weeks.

## Methods

We used data from the Aarhus Birth Cohort. The cohort was established at the Department of Obstetrics at Aarhus University Hospital in August 1991 [30] The obstetrics department was the only maternity unit in the community of Aarhus and served a well-defined geographic area with a population of approximately 250,000 inhabitants. Antenatal care is free of charge in Denmark, and almost all (> 97%) women participated in the programme.

Briefly, all women attending routine antenatal care at Aarhus University Hospital in the period beginning in August 1989 and ending in September 1991 were invited to participate in the study. At enrolment, women at approximately 16 weeks’ gestation were asked to complete a clinical questionnaire providing information about their medical and obstetrics histories, as well as information about their ages, heights, pre-pregnancy weights, levels of alcohol consumption, and smoking habits, as well as a second questionnaire (the research questionnaire) about socio-demographic characteristics and leisure time physical activities [31] The clinical questionnaire provided information included in each pregnant woman’s obstetrical records. Following delivery, the attending midwife completed a structured birth registration form with information about the course of delivery and the condition of the newborn.

The present follow-up study is based on children from the cohort who were born between January 1990 and June 1992. During this period, 8643 women completed the clinical questionnaire, and 6782 women completed both questionnaires. Women who experienced foetal death (*n* = 2), delivered twins and triplets (*n* = 165) and delivered singletons without a valid gestational age (*n* = 15) were excluded, resulting in a study population of 6600 women.

In 2001, **(**January – December), the parents of these children were identified using the civil registration system [32], and they were invited to complete a parent-administered questionnaire concerning their child’s behaviour, physical activity, health, and development. A total of 5147 questionnaires were returned. From this population we excluded children whose parents did not provide information on sports activities per week (*n* = 78). Children with significant physical handicaps, chromosome abnormalities, autism, Asperger syndrome, cerebral palsy, mental retardation, severe chronic diseases, blindness, severe hearing loss and deafness, and severe heart defects were also excluded (*n* = 113) as well as children born before 32 completed gestational weeks (*n* = 15), leaving 4941 singleton infants born at term and moderately preterm, available for analysis.

### Exposure

Moderately preterm delivery was defined as delivery between 32 and 36 completed gestational weeks. Gestational age was estimated by ultrasound measurements before 21 completed weeks of gestation in 86% of cases. In 8% of cases, gestational age was estimated using the date of a valid last menstrual period (LMP), corrected to a cycle length of 28 days: in 6%, gestational age was estimated by the midwife using a less reliable LMP (primarily an LMP without information regarding specific cycle length) or ultrasound measurements taken after 21 weeks [33].

### Outcome

Information regarding each child’s leisure time physical activity level was obtained from the parent-administered questionnaires completed when each child was between 9 and 11 years of age. They were asked how many times per week their child participated in sports activities outside of school, how many hours a week after school their child was active outdoors, how many hours a week their child watched television, how many hours a week (in addition to schoolwork) their child read, including comic books, and how many hours a week (in addition to schoolwork) the child used a computer or played videogames. We created a category for children with very low physical activity levels whose parents reported that they did not participate in sports activities outside of school and engaged in fewer than four weekly hours of outdoor activities. The following characteristics were categorized as described in Table 1: whether the child’s parents cohabited; maternal pre-existing chronic maternal disease; maternal smoking before, during and after pregnancy; partner’s smoking during and after pregnancy; maternal alcohol consumption during pregnancy; maternal leisure time physical activity during pregnancy; and parental education level.

**Table 1:** Parental and offspring (*N* = 4941) characteristics at birth and 9-11 years after moderately preterm birth (32-36 completed weeks). The Aarhus Birth Cohort, Denmark 1989-2001

	Moderately preterm birth n^a^ (%)	Term birth n (%)	*p*-value
*Total*	158 (3.2)	4783 (96.8)	
*Prenatal maternal and paternal factors*			
Cohabiting *Missing data (67)*	151 (96.2)	4583 (97.2)	0.46
Pre-existing chronic disease *Missing data (173)*	13 (8.5)	179 (3.9)	0.01
Smoking before pregnancy *Missing data (11)*	85 (53.8)	1857 (38.9)	< 0.001
Smoking during pregnancy *Missing data (96)*	66 (42.3)	1311 (28.0)	< 0.001
Partner smoking during pregnancy *Missing data (96)*	81 (63.3)	1875 (47.5)	0.001
Alcohol during pregnancy (units per week)			0.99
< 1	94 (59.9)	2786 (59.5)	
1-2	43 (27.4)	1287 (27.5)	
3+ *Missing data (98)*	20 (12.7)	613 (13.1)	
Leisure time physical activity during pregnancy			0.005
Sedentary	80 (52.6)	1867 (40.9)	
Light activity	68 (44.7)	2382 (52.2)	
Moderate to heavy activity *Missing data (225)*	4 (2.6)	315 (6.9)	
Offspring at 9-11 years			0.52
Girl	74 (46.8)	2379 (49.7)	
Boy	84 (53.2)	2404 (50.3)	
Height, mean (SD)	142 (8.1)	142 (8.0)	0.74
Weight, median (min-max) *Missing data (0)*	35 (7.3)	34.5 (7.1)	0.34
Asthma diagnosed by a medical doctor			< 0.001
Yes	30 (19)	399 (8.3)	
No	127 (80.4)	4348 (90.9)	
Do not know *Missing data (0)*	1 (0.6)	36 (0.8)	
Maternal smoking after delivery *Missing data (12)*	85 (53.8)	1926 (40.6)	0.001
Paternal smoking after delivery *Missing data (149)*	95 (61.3)	2063 (44.5)	< 0.001
Maternal professional education			0.07
No education (after secondary schools +/− high schools)	12 (7.6)	354 (7.5)	
Currently studying	10 (6.3)	104 (2.2)	
1-2 years of education (after secondary schools +/− high schools)	44 (27.8)	1295 (26.4)	
3-4 years of education (after secondary schools +/− high schools)	75 (47.5)	2271 (47.9)	
4 + years of education (after secondary schools +/− high schools) *Missing data (40)*	17 (10.8)	763 (16.1)	
Paternal professional education			0.022
No education (after secondary schools +/− high schools)	25 (16.3)	487 (10.5)	
Currently studying	1 (0.7)	64 (1.4)	
1-2 years of education (after secondary schools +/− high schools)	19 (12.4)	553 (12.0)	
3-4 years of education (after secondary schools +/− high schools)	79 (51.6)	2163 (46.8)	
4 + years of education (after secondary schools +/− high schools) *Missing data (167)*	29 (19.0)	1354 (29.3)	

^a^Total differs due to missing values

The children’s heights and weights were described by a mean and SD (standard deviation). The parents were asked if a medical doctor had ever diagnosed their child with asthma.

### Statistical analysis

An initial Chi-square test for independence within contingency tables was used. Testing for trends and t-tests were carried out where appropriate. In a multivariate logistic regression analysis, the relation between preterm birth (gestational age, 32-36 completed weeks gestation vs. ≥ 37 weeks gestation) and physical activity levels in children aged 9 to 11 was tested. A priori, we chose to include the following covariates in the analysis: maternal leisure time physical activity during pregnancy, maternal education level, paternal education level, maternal and paternal smoking habits after delivery, and the sex of the child, given previously demonstrated associations between preterm delivery as well as the child physical activity level [19, 34]. We repeated all analyses in a sub analysis comparing the children born 32-36 weeks to those born 39-40 weeks. Furthermore, we repeated all analyses stratified by sex and after excluding children with asthma. We presented adjusted odds ratios with 95% confidence intervals (CIs). Statistical significance was defined as a two-sided *P* value less than 0.05. We used SPSS 20.0 software (IBM) for all statistical analyses.

## Results

Table 1 shows the proportion of children born moderately preterm and at term in relation to prenatal, parental and offspring characteristics at birth and 9 to 11 years later. In total, 158 infants (3.2%) were born moderately preterm. Moderately preterm birth was associated with pre-existing maternal chronic diseases; maternal smoking before, during and after pregnancy; paternal smoking during pregnancy; less maternal leisure time physical activity during pregnancy and fewer years of parental education. Furthermore, children born moderately preterm had the same height and weight by ages 9 to 11 as children born at term. Children born moderately preterm had a higher frequency of asthma, as 19% of moderately preterm children had asthma compared to 8.3% of children born at term (Table 1).

In our study population, 82% (*n* = 4069) of children participated in sports outside of school on a weekly basis. Only 28% of school-aged children in our population participated in sports activities 3 times per week or more, as recommended by the national health guidelines for children [29]. More than half of the children reported participating in outdoor activities for more than 7 h a week. Eleven percent of the children reported participating in sedentary activities for 7 h or less per week. Less than half of the children reported participating in 15 h or more of sedentary activities per week (Table 2). No differences were observed between children born moderately preterm and children born at term.

**Table 2:** Moderately preterm birth (32-36 completed weeks) and levels of sports activity, outdoor activity and sedentary activity at 9-11 years of age. The Aarhus Birth Cohort, Denmark 1989-1991, N = 4941

	Moderately preterm birth n (%) 158 (3.2)	Total n^a^ 4941	OR	OR 95% CI
Participating in sports during the week				
No	29 (3.3)	872	1.00 (ref)	1.00 (ref)
Yes	129 (3.2)	4069	0.95	1.03 (0.67-1.58)
Sports activity: number of days per week				
0	29 (3.3)	872	1.00 (ref)	1.00 (ref)
1	29 (2.6)	1116	0.78	1.26 (0.60-2.65)
2	51 (3.3)	1559	0.98	1.02 (0.49-2.16)
3	36 (3.8)	947	1.15	1.37 (0.68-2.75)
≥ 4	13 (2.9)	447	0.87	1.63 (0.80-3.35)
Outdoor activity: hours per week				
0-3	23 (2.8)	812	1.00 (ref)	1.00 (ref)
4-6	37 (3.2)	1162	1.13	1.01(0.59-1.74)
7-13	55 (3.2)	1704	1.14	1.02 (0.61-1.70)
14-20	27 (3.3)	830	1.15	0.93 (0.52-1.67)
21-60	7 (3.4)	207	1.20	0.88(0.35-2.22)
Children with very low level physical activity levels				
Yes	6 (2.4)	247	1.00 (ref)	1.00 (ref)
No	152 (3.2)	4694	1.34	1.36 (0.59-3.2)
Sedentary activities: hours per week^b^				
0- 7	14 (2.5)	569	1.00 (ref)	1.00 (ref)
8-14	73 (3.5)	2083	1.44	1.35 (0.74-2.49)
15-21	50 (3.5)	1412	1.46	1.38 (0.73-2.62)
22-28	11 (2.0)	549	0.81	0.68 (0.30-1.57)
29+	6 (2.6)	233	1.05	0.81 (0.28-2.36)

^a^ Total differs due to missing values

^b^ Watching television, using a computer, playing video games and reading books and comics

*OR* Odds ratio, *CI* Confidence interval

The adjusted multivariate analysis showed that children born moderately preterm participated in sports activities as often as their peers born at term (OR = 1.03, 95% CI (0.67-1.58)) and participated in sports activities 3 times per week and ≥ 4 times per week as often as their peers born at term (Table 2). Similarly, children born moderately preterm played outside as often as their peers born at term. Furthermore, children born moderately preterm had the same level of very low physical activity as their peers born at term (OR = 1.36, 95% (0.59-3.2)). Multiple logistic regression analyses showed that infants born moderately preterm participated in sedentary activity (watching television, using a computer, playing videogames, reading books and comics) as often as their peers born at term (Table 2). Sub-analyses showed similar results when we compared the children born moderately preterm with the children born in weeks 39-40.Additional sub-analyses showed that results were similar for boys and girls and that the results remained essentially unchanged after excluding 429 children diagnosed with asthma (data not shown).

## Discussion

Due to the well-established higher risk of physical disadvantages, one may expect that children born preterm would be less leisure time physically active than their peers born at term. However, our data showed no differences in the activity levels of term and moderately preterm children aged 9 to 11 years. Parents of children born moderately preterm reported that their children were involved in leisure time sports activities to the same extent as children born at term. The same proportion of children in both groups followed the recommendation of high intensive physical activity by the Danish National Board of Health [29].

Additionally, there were no differences between children born moderately preterm and children born at term, considering hours spent participating in outdoor activities per week. Nor were there any differences in hours spent participating in sedentary activities—watching television, playing videogames, using a computer and reading.

Previous studies of physical activity and the exercise capacity of preterm children showed diverging results. Consistent with the results of our study, some studies of preschool- and school-aged children reported no differences in the levels of physical activity of preterm and term children [9, 35–37]. Other studies showed that young adults who were born preterm reported less exercise than control subjects [19, 38, 39]. This disparity may reflect real differences between the populations studied but may also be due to differences in the measurements of physical activity used for each of the studies. In our study, we measured each child’s leisure time physical activity level based both on the frequency of sports activities per week and hours of outdoor activities per week, but we did not measure the performance level of each child.

Most previous studies included only children with very low birthweights (1000-1500 g) or extremely low birthweights (< 1000 g) [9, 17–19, 35, 37, 38]. This may increase the likelihood of observing differences in the activity levels of children born preterm and children born at term due to the much higher overall morbidity observed in this newborn population [9]. However, moderately preterm infants account for 80% of premature births [17]. Therefore, it is important to focus on this large group, particularly because there is increasing evidence that these patients are also at increased risk for developmental and behavioural morbidities [17, 18]. To the best of our knowledge, our study is the first to investigate a group of moderately preterm infants regarding leisure time physical activity.. By excluding multiple births and all children with handicaps that may influence their ability related to physical activity our results can only be generalized to singletons without a moderate to severe physical handicap born moderately preterm.

Our moderately preterm population had a significantly higher prevalence of asthma (19%) than children born at term (8.3%), which has also been demonstrated in other populations [40]. However, there was no difference in the amount of time spent engaged in physical activity. One explanation may be that the parents of children born preterm who suffer from asthma may be more motivated to enrol their child in sports activities. Another reason could be that most Danish children have well controlled asthma or that parents with children born preterm reported that their children suffer from asthma more often than other parents. The results of our sub-analysis demonstrated that the results remained essentially unchanged after excluding children diagnosed with asthma.

We conducted a population-based cohort study using prospective data from a single prenatal care setting and obstetrics department in the second largest city in Denmark, where prenatal care and hospital delivery are free of charge; therefore, the cohort represents all socio-demographic groups. We excluded children with severe mental and physical health problem, twins and triplets to enhance comparability between the two groups of children. A self-administered questionnaire was used to collect information about the children’s physical activities; this could potentially introduce inaccuracy. The questionnaire used to assess physical activity resulted in a mere description of duration and type of leisure time activity rather than intensity. The type and duration should be simple for parents to reconstruct since many children attend sports on specific weekdays for a well-defined number of hours that the parents will pay for. Differential misclassification of children’s physical activity would impact our results. However, we have no reason to believe that parents of preterm children would report their activity differently than parents of children born at term. Another way to measure physical activity is with an objective form of measurement, such as an accelerometer, as seen elsewhere [9]. Objective measurements may introduce selection biases into the study due to compliance problems while obtaining measurements or due to a lack of willingness to participate in the assessment. The care of children born after 32 weeks has essentially remained unchanged over the last decades. Accordingly, we believe that our results may be extrapolated to more recent time.

The fraction of moderately preterm children was similar in children followed up to that among children lost to follow up (3.2% vs 4.2%). However, if the families with more physical active children were more prone to participate if the child was preterm than if they were term, this may have introduced bias towards a more favorable physical profile for preterm compared to term children. However, we consider this explanation for our results unlikely.

The proportion of healthy children born moderately preterm among participants is comparable to the proportion of preterm delivery in the Danish national birth register at that time [41]. This indicates that selection biases may not be a major issue. The overall rate of preterm delivery in Denmark is rather low compared to other industrialized countries such as United States [42]. However, we have no reason to believe that the results of this study cannot be found in populations in other countries.

Physical inactivity is associated with a higher risk of obesity, and children and adolescents are becoming more sedentary [21, 22]. It may be even more important for infants born preterm to follow the guidelines for physical activity, as studies have indicated that preterm delivery is associated with a higher risk of stroke [43] and type 2 diabetes [24] in adulthood. These diseases are associated with lower levels of physical activity, and there is evidence that physical activity may prevent them [21, 22].

## Conclusions

In this study of 9- to 11-year-old children, we found no differences in leisure time activities as weekly number of sports activities outside school, hours spent on outdoor activity and hours spent on sedentary activity between children born moderately preterm and children born at term. This finding is reassuring as physical activity is a fundamental element of a normal childhood and is important for long-term development [44]. Only one-third of school-aged children regardless of gestational age at birth in our study were as physically active as recommended by the national health guidelines for children [29]; thus, a focus on the factors that increase physical activity of all children is necessary.

## Abbreviations

CI: Confidence intervals
LMP: Last menstrual period
OR: Odds Ratio
SD: Standard deviation
